# Proteomic Profiling of Alveolar Macrophages Identifies Loss of Lysosomal Content as an Indicator of Nanofiber‐Induced Frustrated Phagocytosis

**DOI:** 10.1002/smll.202510530

**Published:** 2026-01-17

**Authors:** Tobias Stobernack, Antje Vennemann, Carla Ribalta, Julia Schendel, Oliver Gräb, Rico Ledwith, Mario Pink, Andrea Haase, Martin Wiemann, Verónica I. Dumit

**Affiliations:** ^1^ Department of Chemical and Product Safety German Federal Institute for Risk Assessment (BfR) Berlin Germany; ^2^ IBE R&D Institute for Lung Health gGmbH Münster Germany; ^3^ University of the Western Cape (UWC) Department of Chemical Sciences Bellville South Africa; ^4^ Federal Institute for Occupational Safety and Health (BAuA) Materials and Particulate Hazardous Substances Berlin Germany; ^5^ Freie Universität Berlin Institute of Pharmacy Berlin Germany

**Keywords:** fiber pathogenicity paradigm (FPP), Mitsui‐7, NR8383, silicon carbide, toxicity

## Abstract

Toxicological research on inhalable fibers, such as asbestos, has identified material morphology (i.e., length and diameter) and bio‐persistence as drivers of adverse health effects (e.g., fibrosis, lung cancer, mesothelioma). Although nanofibers may meet these criteria, their small diameters may enable them to adopt different shapes, affecting their toxicity. While nanofiber pathogenicity is still assessed using animal models, the development of alternative in vitro methods relies on a mechanistic understanding of toxicity. Here, we address nanofiber‐induced protein changes in alveolar macrophages by analyzing whole cell lysates and supernatants of NR8383 cells exposed to silicon carbide nanofibers, Mitsui‐7 carbon nanotubes, and Printex‐90. While all materials elicited a similar dose‐dependent cytotoxicity, there was a nanofiber‐specific release of TNF‐α and glucuronidase. Proteomic profiling after treatment with low, non‐cytotoxic concentrations confirmed the inflammatory response and revealed a release of 20 lysosomal, luminal hydrolases, including six cathepsins, into the extracellular supernatant. In cell lysates, these hydrolases were decreased, while membrane‐associated lysosomal proteins remained unchanged, suggesting that macrophages engulfing long nanofibers release lysosomal content from open membrane pouches during frustrated phagocytosis. Additionally, 17 biomarkers of nanofiber‐induced toxicity were identified as potential targets for predictive, animal‐free screening. These early markers may be of value for assessing nanofiber toxicity.

## Graphical abstract

This proteomic study examines how nanofibers affect lung macrophages by analyzing protein changes following exposure to various fibrous materials. Nanofibers triggered the release of inflammatory enzymes, and importantly, lysosomal content release was identified as an early event associated with frustrated phagocytosis. Seventeen sensitive and relevant protein biomarkers were identified, offering potential for animal‐free methods to predict nanofiber toxicity.

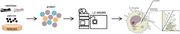

## 1 Introduction

High‐aspect‐ratio materials such as nanofibers (NFs) are increasingly used across different sectors, including energy storage, thermal and electrical transport, water treatment, and biomedical technologies, among others [1, 2, 3, 4], prompting concerns about their potential health effects upon inhalation. However, the responsible use of NFs critically depends on their comprehensive safety evaluation. Toxicological research on fibers such as asbestos has identified both material morphology (length and diameter) and bio‐persistence, as the main drivers of the adverse health effects following inhalation exposure. These effects include fibrosis, lung cancer, and mesothelioma [5, 6]. This understanding led to the development of the Fiber Pathogenicity Paradigm (FPP) and shaped the criteria established by the World Health Organization (WHO), which set the criteria for critical fibers as a minimum length of 5 µm, a respirable diameter not exceeding 3 µm, and an aspect ratio of 3:1 or higher [7]. Fibers meeting these criteria may potentially cause cancer, provided they are inhalable and sufficiently bio‐persistent. The accepted mechanism for fiber toxicity involves frustrated phagocytosis, wherein a macrophage attempts to engulf a fiber [8]. However, when the fiber's length exceeds the length of the immune cell, the process is impaired. Consequently, the macrophage initiates inflammatory responses, which in turn recruit further immune cells, leading to chronic inflammation [9, 10, 11]. Considering that inflammation is a reversible process, the downstream molecular events that convert an acute inflammatory response into persistent tissue damage and carcinogenesis remain poorly understood. There is a knowledge gap between general inflammatory responses and those specifically triggered by high‐aspect‐ratio fibers. It remains unclear whether frustrated phagocytosis initiates unique pathways that drive pathogenic outcomes.

Although NFs meet the criteria, they challenge the FPP, as their small diameters grant the NFs flexibility, diminishing their rigid fiber‐character [12]. Thin NFs can tangle and form particle‐like shapes, affecting their toxicity [13]. For instance, multi‐walled carbon nanotubes (MWCNTs) with diameters smaller than 30 nm tend to agglomerate into particle‐like structures [14], and their toxicity appears to correlate with that observed for bio‐persistent granular particles [15]. In contrast, rigid MWCNTs, like Mitsui‐7, with a mean diameter of ca. 50 nm, have shown evidence of lung carcinogenicity in rat studies [16], genotoxicity [17], and profibrotic response in human lung cells [18].

The development of a testing strategy for NFs remains urgently needed to ensure the safe and sustainable use of rapidly emerging novel NFs. By now, the assessment of fiber pathogenicity relies on animal testing in a case‐by‐case approach, which raises both ethical and scientific concerns. The major hurdle in using rodents for fiber toxicity assessments is the long latency periods prior to cancer development [19]. Additionally, the European Commission has a long‐standing policy in line with the 3R (replace, reduce, and refine) principles, aiming at phasing out animal testing in the area of chemical safety assessment [20]. Therefore, advancing in the development of new approach methodologies (NAMs) that meet ethical standards and ensure reliable hazard evaluations are key to reach this goal, but critically depends on the comprehensive understanding of the underlying toxicity mechanisms [21]. To interpret long‐term toxicological effects, the Adverse Outcome Pathways (AOPs) provide a framework [22], which connects a molecular initiating event to the adverse outcome by intermediate key events at different biological levels [23, 24]. Currently, there are only a few AOPs addressing fiber toxicity, among them, pulmonary fibrosis (AOP173) [25], frustrated phagocytosis‐induced lung cancer (AOP303) [26], and malignant mesothelioma (AOP409) [27]. Despite the advanced state of these AOPs, knowledge gaps still exist. To this end, omic techniques can contribute significantly to the AOP substantiation by providing insights into early cellular responses [22, 28, 29]. Additionally, the identification of biomarkers that are relevant exclusively to frustrated phagocytosis is key for the development of NAMs to assess NF toxicity.

In a previous study on cell lysates from NR8383 cells, we had already identified NF‐specific protein changes comprised in a proteomic preliminary fingerprint by comparing ground and intact NFs from silicon carbide (SiC) and titanium dioxide (TiO_2_) [30]. Notably, lysosomal luminal proteins in cell lysates exhibited a significant decrease in abundance, including several hydrolases, such as cathepsins. Since their specific role and localization remained unclear, the present work aimed at elucidating the mechanistic involvement of the identified proteins in NF‐induced morphological toxicity. We hypothesized that the lysosomal content is released to the extracellular matrix when the macrophages are in contact with critical NFs, as inferred from a conceptual illustration on frustrated phagocytosis by Schinwald & Donaldson (2012) [31]. To this end, proteomic analyses were conducted on cell lysates and culture supernatants from NR8383 rat alveolar macrophages exposed to Mitsui‐7, its particulate counterpart Printex‐90, and SiC NF as reference material. A second objective of the work was to determine if the preliminary fingerprint is applicable to carbon‐based materials and can be refined to the most sensitive, reliable, and relevant protein markers of NF toxicity.

## 2 Results and Discussion

### 2.1 Material Characterization

The characterization of examined nanomaterials (NMs) in this study is summarized in Table 1 and is based on results from Jackson et al. (2012) [32], Porter et al. (2010) [33], and the InnoMat.Life project [34]. Dispersion of the NMs was performed via sonication, adhering to the NANoREG D5.07 SOP 04 standard operating procedure [35].

**TABLE 1:** Material characterization.

Material	Manufacturer	Mean Diameter (nm)	Mean Length	BET (m^2^/g)	Main impurities (% (w/w))	Shape
Printex‐90 [32]	Orion (Degussa), Germany	14	14 nm	310	Organic impurity content <1%. 0.8% N, 0,01% H_2_	Spherical particle
SiC nanowire [30, 34]	ACS materials (NWSC0202)	190	11,7 µm	—	<0.11 CaO, <0.08 TiO_2_, <0.1 Al_2_O_3_, <0.08 Fe_2_O_3_, <0.07 MgO <0.06 Na_2_O, <0.05 ZrO_2_	Rigid nano/micro‐fiber, 56% WHO fraction
Mitsui‐7 [33]	Mitsui & Co Japan. Donated by NRCWE JRCNM40011a Lot:05072001K28	49 ± 13.4	3.86 ± 1.94 µm	26	0.41% Na, 0.32% Fe	Rigid wall (needle like)

### 2.2 Toxicity Screening with Alveolar Macrophages

To evaluate the cellular effects of NFs, the alveolar macrophage assay based on NR8383 cells was used. This cell line showed high accuracy in predicting short‐term inhalation toxicity of NMs [36], and a high sensitivity to differentiate effects of NF from those of their particulate counterparts [30]. Importantly, NR8383 cells allow particle administration and uptake under serum‐free conditions [36], enabling the easy collection of proteins secreted by the cells in response to the treatment for proteomic analysis. This supports the straightforward use of the assay for studying exposure‐related changes in the secretome without the interference of serum proteins.

**FIGURE 1:**
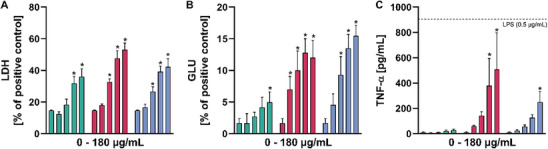
Toxicity screening with the alveolar macrophage assay. Printex‐90 (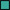), SiC nanofibers (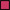), and Mitsui‐7 (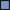) were administered to NR8383 macrophages (22.5, 45, 90, and 180 µg/mL) under serum‐free conditions for 16 h. Resulting cell culture supernatants were assayed for (A) lactate dehydrogenase (LDH), (B) glucuronidase (GLU), and (C) TNF‐α. For (C) LPS (0.05 µg/mL) was used as a positive control, and its readout is indicated with a dashed line. Results are means ± standard deviation from three biological replicates. A two‐way ANOVA (*p* ≤ 0.05) Dunnett was used to evaluate significant differences between the samples.

**FIGURE 2:**
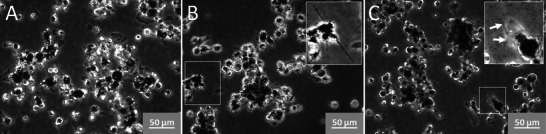
Phase contrast micrographs of NR8383 rat alveolar macrophages exposed to 22.5 µg/mL of either test material, as used for proteome experiments: (A) Printex‐90, (B) SiC NFs, and (C) Mitsui‐7 NFs. Note that material loadings are comparable and largely complete, except for some long SiC fibers protruding from the cells and some Mitsui‐7 fibers seen among the cells. Inserts in B and C highlight selected cells (marked by white frames) contacting long fibers in the process of frustrated phagocytosis. White arrows in C point to a fiber structure attached to a cell.

The release of lactate dehydrogenase (LDH), glucuronidase (GLU), and tumor necrosis factor‐alpha (TNF‐α) is shown in Figure 1. NR8383 cells were incubated with increasing concentrations of Printex 90, SiC, and Mitsui‐7. All materials elicited a release of LDH, indicating a dose‐dependent cytotoxicity, whereby membrane damage became significant at 45–90 µg/mL. The activity of the lytic enzyme GLU was similarly increased in the supernatant. Interestingly, the SiC and Mitsui‐7 NF induced a more pronounced response (Figure 1A,B) suggesting a release mechanism different from the mere membrane damage. TNF‐α was measured as an indicator of the pro‐inflammatory properties. Again, a dose‐dependent increase of TNF‐α upon treatment with SiC and Mitsui‐7 NF was observed, but not upon Printex‐90 treatment (Figure 1C). The results from the alveolar macrophage assay revealed 22.5 µg/mL of any material to be a non‐cytotoxic concentration suited for proteomic profiling of cell lysates and cell culture supernatants. Using a non‐cytotoxic fiber concentration is essential to prevent contamination by nonspecific cellular proteins or their fragments, thereby ensuring the identification of proteins specifically associated with frustrated phagocytosis. At this concentration, Printex‐90, SiC NFs, and Mitsui‐7 appeared as prominent dark inclusions concentrated in many cells (Figure 2), whereby SiC NFs and a few Mitsui‐7 agglomerates were seen protruding from some cells (Figure 2C), which is typical for frustrated phagocytosis. However, since no major portion of any material remained outside the cells, the uptake of all materials was deemed close to complete.

**FIGURE 3:**
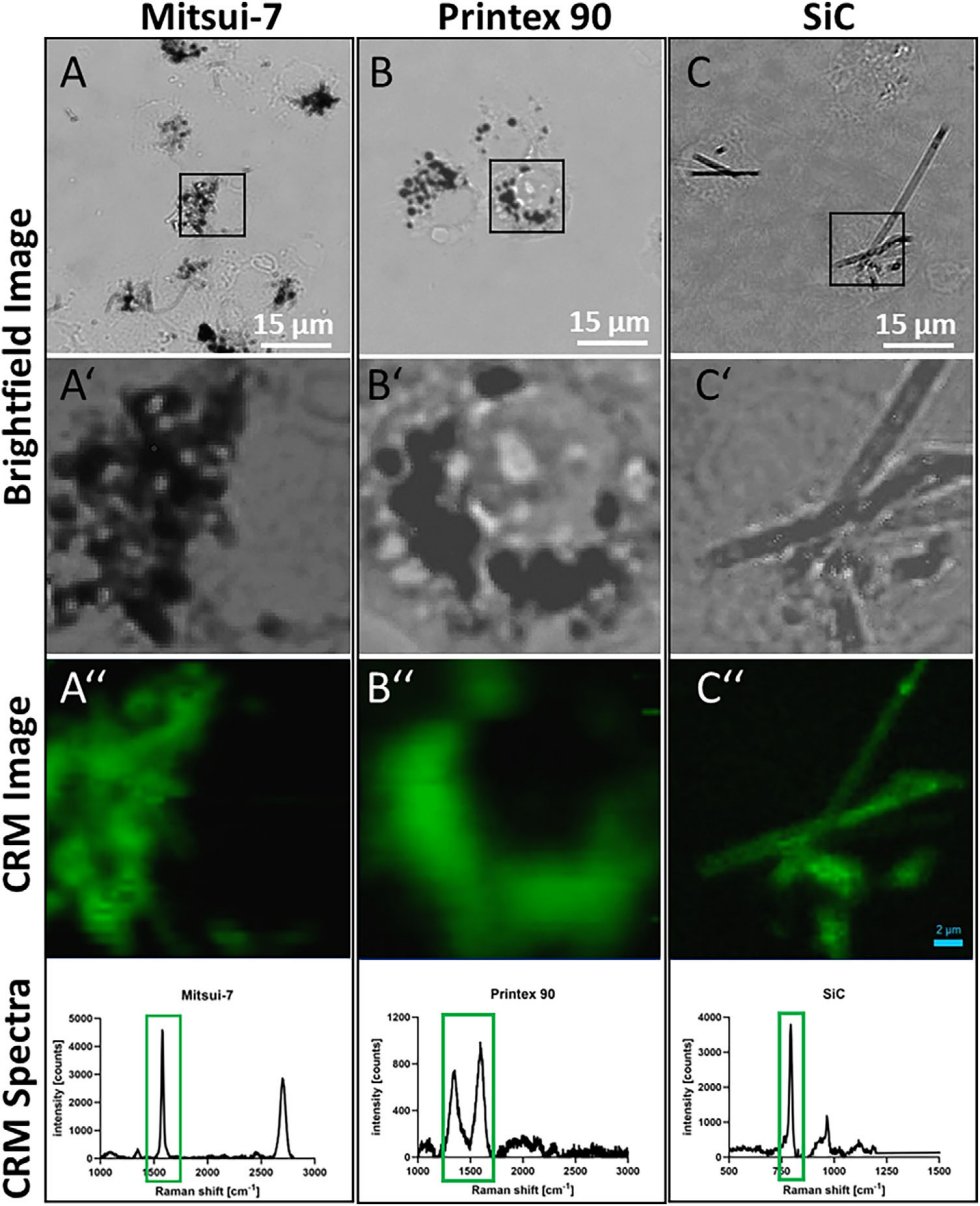
Identification of test materials in NR8383 macrophages by Confocal Raman Microscopy (CRM). Bright field images of formalin‐fixed cytospin preparations of cells loaded with either 22.5 µg/mL Mitsui 7 (A), Printex 90 (B) and SiC Nanofibers (C); (A′–C′) show the regions of interest boxed in (A–C); (A″–C″) show the Raman spectra with characteristic bands, as outlined in the text (green boxes); these signals (in green) are co‐localized with the dark inclusion inside cells (compare A′–C′) confirming their chemical nature.

Carbon‐based materials have characteristic Raman spectra that reflect the structure of the bonded carbon. Here we used confocal Raman microscopy, especially to detect Carbon Black and Mitsui‐7 NFs in macrophages [37]. Mitsui‐7, like all MWCNTs, shows Raman bands in the spectral range of 1350 cm^−1^ (D‐band), around 1580 cm^−1^ (G‐band), and close to 2700 cm^−1^ (2D‐band) [38]. Printex‐90, which consists of amorphous carbon, leads to broader Raman bands at 1350 cm^−1^ and 1600 cm^−1^ [39]. SiC has a characteristic Raman band at 780 cm^−1^ by which it could be identified [40]. As shown in Figure 3A–C, all aforementioned Raman bands were detectable in cells prepared on glass slides by cytocentrifugation, thus confirming the identity and uptake of all materials.

### 2.3 Proteomic Profiling

In a previous study, we had exposed rat alveolar NR8383 macrophages to a concentration of 45 µg/mL SiC and TiO_2_ NFs (i.e., beginning cytotoxicity) and identified a preliminary proteomic fingerprint consisting of 58 potential biomarkers for NF toxicity [30]. In the present study, the same cell line was exposed to Mitsui‐7, a known carcinogenic carbon nanotube, and Printex‐90, its particulate counterpart, to extend the findings to carbon‐based materials. SiC NFs were also included, as the previous study has shown that this material induces fiber‐specific proteomic alterations [30]. In order to identify those biomarkers with the highest sensitivity, the concentration of tested materials was reduced to half that used in the previous study, with exposure levels chosen to induce minimal cytotoxicity (see Figure 1). Non‐cytotoxic concentrations ensure that observed changes reflect early, mechanism‐specific cellular responses rather than secondary effects such as cell death. In addition, to understand the role and localization of found biomarkers, unlike the previous study, proteomics measurements were performed on cell lysates and extended to cell culture supernatants as well.

For proteomic analysis, samples were collected and processed according to a bottom‐up protocol previously described by Stobernack et al. [41]. All samples were generated in biological triplicates and analyzed three times by LC‐MS/MS using data‐dependent acquisition (DDA) mode. Relative protein quantification was performed using Tandem Mass Tag (TMT) labeling for cell lysates, while supernatants were concentrated, and resulting proteins were quantified using label‐free quantification (LFQ). Within the *Rattus norvegicus* proteome 17051 unique peptides corresponding to 3019 proteins were identified with a false discovery rate (FDR) of ≤ 0.01 in the cell lysate, and 6654 peptides corresponding to 1363 proteins in the supernatant. For protein quantification, at least 70% valid values were mandatory among all samples after log_2_ transformation of intensities, enabling the quantification of a maximum of 2098 proteins in the lysates and 848 proteins in the supernatants (Table 2, Supporting Information II: Tables S1 and S2).

**TABLE 2:** Peptides and proteins identified (FDR ≤ 0.01) in NR8383 rat alveolar macrophages, untreated or treated with 22.5 µg/mL of Printex‐90, SiC NFs, or Mitsui‐7 for 18 h. Protein levels under treatment were quantified relative to untreated controls using LC‐MS/MS analysis. Each condition was applied to three biological replicates, and each resulting sample was measured three times by LC‐MS/MS.

Location	ID	Quantifiableproteins	Alteredproteins ^a^	Identifiedpeptides
Lysate	control	2098	—	17501
	Printex‐90	2098	78	17501
	SiC NF	2098	201	17501
	Mitsui‐7	2098	286	17501
Supernatant	control	848	—	6654
	Printex‐90	848	61	6654
	SiC NF	848	133	6654
	Mitsui‐7	848	313	6654

^a^ in comparison to control (FDR ≤ 0.05, t‐test, s_0_ = 0.1)

**FIGURE 4:**
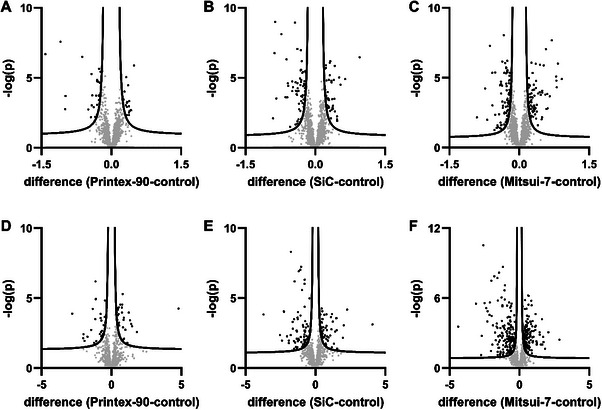
Volcano plots showing the proteins identified (FDR = 0.05, s_0_ = 0.1, t‐test) in NR8383 rat alveolar macrophage lysates (top) and culture supernatants (bottom) following treatment (22.5 µg/mL, 18 h) with Printex‐90 (A,D), SiC nanofibers (B,E), or Mitsui‐7 (C,F). Proteins exhibiting significant changes are marked in black. Each treatment was performed in three biological replicates, and every sample was analyzed in triplicate by LC‐MS/MS.

To identify proteomic alterations, volcano plots were generated (t‐test, FDR ≤ 0.05, s_0_ = 0.1), comparing the different treatments to untreated controls (Figure 4, Supporting Information II: S3–S8). A total of 78 proteins exhibited significant changes in their levels in cell lysates following treatment with the particulate NM, Printex‐90, relative to the control. Cells exposed to both NFs showed a greater number of significantly altered proteins. Specifically, treatments with SiC NFs and Mitsui‐7 resulted in 201 and 286 proteins, respectively. A similar trend was observed in the cell culture supernatants of treated samples. In Printex‐90‐treated cells, 61 proteins were significantly altered, while this number increased to 133 and 313 following treatments with SiC NFs and Mitsui‐7, respectively.

Printex‐90 was employed as a particle counterpart for Mitsui‐7, and was expected to induce no fiber‐specific effects. However, Printex‐90 can induce oxidative stress by influencing ROS and calcium homeostasis of A549 lung epithelial cells [42, 43, 44]. Furthermore, Printex‐90 has been shown to elicit pro‐inflammatory responses in mouse lung and liver tissues [45]. Regarding the fibrous materials, Mitsui‐7 has a shorter mean fiber length compared to SiC (see Table 1) and is, therefore, expected to contain fewer critical fibers. Due to the presence of contaminant ions in Mitsui‐7, such as iron, oxidative stress through Fenton reactions was expected [46, 47]. As in our previous study, we used SiC for comparison, albeit at a lower concentration (45.0 vs. 22.5 µg/mL) [30]. Accordingly, fewer proteomic alterations were observed.

#### 2.3.1 Pro‐Inflammatory Response

Inflammation is a critical process addressed by different key events (KE 87, 1496‐7) in AOPs related to fiber toxicity [25, 26, 27]. Inflammation can lead to adverse effects such as cancer, lung fibrosis, and mesothelioma [9, 10, 11]. To evaluate to what extent our proteomic measurements capture any pro‐inflammatory response of NR8383 cells treated with SiC NF and Mitsui‐7, we assessed changes in the levels of proteins associated with lung inflammation encompassed in an ad hoc pathway [48]. This pathway contains 195 proteins and was identified as a highly sensitive tool to reflect proteome changes elicited by rigid carbon nanotube treatment, but not by tangled or particulate carbon materials, as demonstrated in a meta‐analysis of omic datasets [29]. The same set of 195 proteins was previously shown to be affected by higher concentrations of SiC or TiO_2_ NFs [30].

**FIGURE 5:**
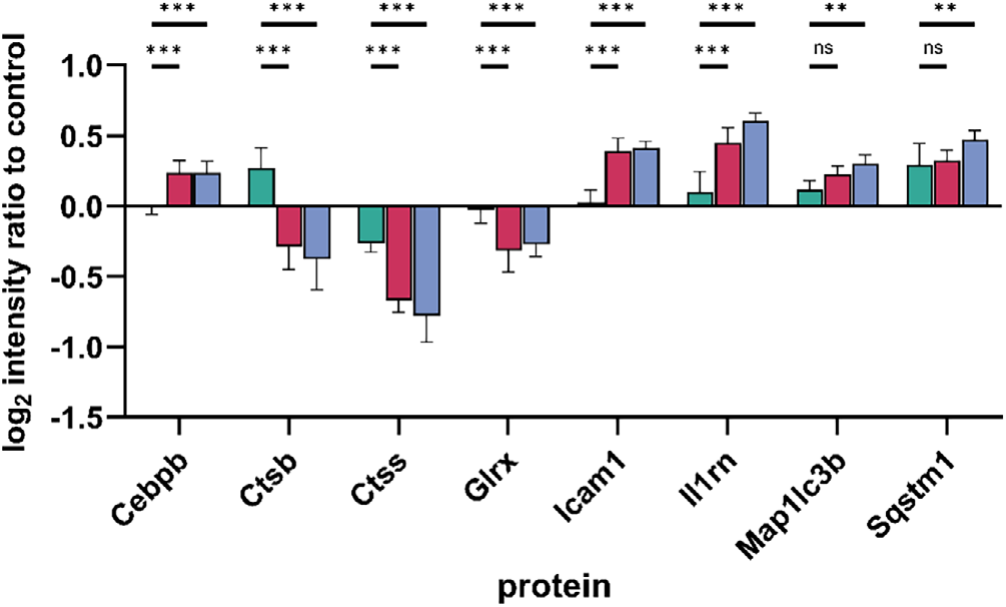
Calculated intensity ratios of significantly altered proteins associated with the lung inflammation pathway by Bahl et al., 2023 [48] relative to control. Proteins were identified in lysates of NR8383 rat alveolar macrophage cells treated (22.5 µg/mL, 18 h) with the nanoparticle Printex‐90 (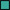), SiC nanofibers (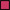), and Mitsui‐7 (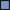). Samples were generated in biological triplicate and measured three times using LC‐MS/MS. Results are expressed as mean values ± standard deviation. Statistical significance was determined by two‐way ANOVA (*p* ≤ 0.05) followed by Dunnett's multiple‐comparison test. Ns (not significant) *p* > 0.05; ^*^ *p* ≤ 0.05; ^**^ *p* ≤ 0.01; ^***^ *p* ≤ 0.001. Cebpb: CCAAT/enhancer‐binding protein beta, Ctsb: cathepsin B, Ctss: cathepsin S, Glrx: glutaredoxin‐1, Icam1: intercellular adhesion molecule 1, Il1rn: interleukin‐1 receptor antagonist protein, Map1lc3b: (Microtubule‐associated protein 1 light chain 3 beta), Sqstm1: sequestosome‐1.

Of the 195 proteins included in the lung inflammation pathway, 31 proteins were identified in our proteomic dataset. Eight of them were significantly altered in their levels in comparison to control (Figure 5), whereby increased levels of Cebpb (CCAAT/enhancer‐binding protein beta), Icam1 (Intercellular adhesion molecule 1), (Interleukin‐1 receptor antagonist), Map1lc3b (Microtubule‐associated protein 1 light chain 3 beta), and Sqstm1 (sequestosome‐1) were accompanied by decreases in Ctsb (cathepsin B), Ctss (cathepsin S), and Glrx (Glutaredoxin‐1).

One role of Ctsb in fiber toxicity has already been described and consists in its release from lysosomes into the cytosol, as a consequence of lysosomal membrane permeabilization. Cytosolic Ctsb then causes activation of the NALP3 inflammasome, thus promoting the cleavage of caspase‐1, which then facilitates the processing and secretion of pro‐inflammatory cytokine IL‐1β [49, 50]. However, a leakage of Ctsb from lysosomes into the cytosol would not explain its reduced levels observed in the proteomics measurements of whole cell lysates (Figure 5). Therefore, other mechanisms such as lowered synthesis, degradation, or release from the cell have to be taken into account (see below).

While we observe a general pro‐inflammatory response to NFs based on the release of TNF‐α (Figure 1) and on the detection of the altered lung inflammation proteins, it is important to note that our proteomic approach cannot detect protein activation states, such as activated NALP3. Additionally, NALP3 is known to be triggered by a variety of other stimuli, including pathogenic microbes [51, 52] (e.g., Mycobacterium tuberculosis [53]), and cellular stress signals, such as ion flux, oxidative stress, and mitochondrial dysfunction [54], and by certain NMs [55]. Therefore, although NALP3 likely contributes to fiber‐induced inflammation, its activation may not be specific to fibers but rather part of a broader, generalized cellular stress response.

#### 2.3.2 KEGG Pathway Analysis

Considering the significant alterations in protein levels following material treatment, a KEGG pathway [56, 57, 58] enrichment analysis was performed on the cell lysates to identify specific biological routes possibly affected by NF treatment. SiC NF and Mitsui‐7 treatments strongly influenced the lysosomal (n = 30 and 35) and metabolic KEGG pathways (n = 41 and 56), whereas the effect of Printex‐90 on these pathways was far lower (lysosome: 8; metabolic: 16, see Supporting Information I: Table S1). All pathway changes in cells treated with SiC NFs and Mitsui‐7 are in agreement with those previously identified in NR8383 cells treated with SiC and TiO_2_ NFs [30]. This not only confirms our previous proteomics analysis but also extends its relevance to the fibrous carbon material Mitsui‐7. Notably, enrichment analysis of cell culture supernatants from NR8383 macrophages treated with Printex‐90 revealed no affected pathways (Supporting Information I: Table S2). In contrast, SiC NF and Mitsui‐7 treatments resulted in the detection of proteins associated with the KEGG pathways ribosome and lysosome (Supporting Information I: Table S2) for cell culture supernatants.

To identify biomarkers of morphology‐dependent toxicity, the proteins that were significantly altered in both SiC NF‐ and Mitsui‐7‐treated cells, but not in Printex‐90‐treated cells, were of particular interest. In total, 124 proteins were identified in lysates of NR8383 cells that were affected in a morphology‐dependent manner, while 89 such proteins were detected in the supernatant. KEGG pathway analysis of these proteins identified the lysosome pathway in both lysates and supernatants as a central response to NF‐exposure (Table 3).

**TABLE 3:** KEGG pathway analysis via the STRING database (FDR≤ 0.01). Analysis is based on proteins significantly altered in NR8383 rat alveolar macrophages in response to separate treatments with SiC nanofibers and Mitsui‐7, but not with Printex‐90, relative to control (22.5 µg/mL, 18 h, n = 3), in both cell lysates and culture supernatants.

Sample type	KEGG pathway affected	Count	FDR
Cell lysate	Lysosome	26 of 120	9.2E‐30
	Metabolic pathways	29 of 1431	1.7E‐07
	Glycosaminoglycan degradation	6 of 18	3.4E‐07
	Tuberculosis	10 of 155	2.2E‐06
	Galactose metabolism	5 of 25	3.9E‐05
	Other glycan degradation	4 of 17	2.7E‐04
	Apoptosis	7 of 122	3.5E‐04
	Carbon metabolism	6 of 112	2.0E‐03
	Cholesterol metabolism	4 of 48	7.1E‐03
Supernatant	Lysosome	10 of 120	5.6E‐08

### 2.4 Refining the Preliminary Fingerprint, Predictive of Nanofiber Toxicity

In our previous proteomic study, treatment of NR8383 cells with SiC and TiO_2_ NFs revealed a substance‐independent cellular response, and a set of 58 proteins was selected for a preliminary proteomic fingerprint to indicate morphology‐driven NF effects. In the present study, with reduced NFs concentration, 41 of the 58 previously identified fingerprint proteins were detected in cell lysates (Supporting Information I: Figure S1, Supporting Information I: Table S3). Based on changes in protein abundance, 17 proteins out of the detected 41 proteins showed the most consistent and significant alterations and, therefore, were selected for a refined fingerprint with increased sensitivity (Table 4).

**TABLE 4:** Refined proteomic signature associated with morphology‐dependent toxicity identified in NR8383 rat alveolar macrophage lysates.

#	Uniprot ID	Protein names	Abbrev.	Location	Function
1	P30919	Aspartylglucosaminidase	Aga	LL	Hydrolase: asparaginase
2	Q6AYS3	Cathepsin A	Ctsa	LL ^a^	Hydrolase: protease
3	P00787	Cathepsin B	Ctsb	LL	Hydrolase: protease
4	P80067	Cathepsin C	Ctsc	LL	Hydrolase: protease
5	Q02765	Cathepsin S	Ctss	LL	Hydrolase: protease
6	Q9R1T3	Cathepsin Z	Ctsz	LL	Hydrolase: protease
7	Q6P7A9	Lysosomal alpha‐glucosidase	Gaa	LL	Hydrolase of glycogen
8	Q32KJ5	N‐acetylglucosamine‐6‐sulfatase	Gns	LL	Hydrolase of glycosaminoglycans
9	Q6AY20	Cation‐dependent mannose‐6‐phosphate receptor	M6pr	LM	Targeting of proteins to the lysosome
10	Q66H12	Alpha‐N‐acetylgalactosaminidase	Naga	LL	Hydrolase of glycoproteins and glycolipids
11	F7FJQ3	NPC intracellular cholesterol transporter 2	Npc2	LL	Cholesterol transporter
12	P45479	Palmitoyl‐protein thioesterase 1	Ppt1	LL	Hydrolase of thioester bonds in palmitoylated proteins
13	P11345	RAF proto‐oncogene serine/threonine‐protein kinase	Raf1	C	regulatory link between the membrane‐associated Ras GTPases and the MAPK/ERK cascade
14	P25086	interleukin‐1 receptor antagonist protein	Il1rn	S	Anti‐inflammatory antagonist of interleukin‐1
15	O08623	sequestosome‐1	Sqstm	C	Bridge between polyubiquitinated proteins and autophagosomes
16	Q00238	intercellular adhesion molecule 1	Icam1	M	Leukocyte adhesion
17	Q9ESH6	glutaredoxin‐1	Glrx	C	Reduces low molecular weight disulfides and proteins

LL: lysosomal lumen.

LM: lysosomal membrane.

C: cytoplasm.

S: secreted.

M: membrane.

^a^ 10% membrane associated [59].

Worth noting, Ctsb and Ctss belong to both the lung inflammation pathway and the KEGG lysosome pathway. Interestingly, the majority of proteins in the refined fingerprint are lysosomal hydrolases, in line with the KEGG pathway analysis (Table 3), which pointed out this pathway to be affected in whole cell lysates and supernatants. This provides further support for the initial hypothesis that lysosomal proteins are key actors in the mechanism underlying morphology‐driven NF toxicity.

### 2.5 Lysosomal Proteins in Nanofiber Toxicity

**FIGURE 6:**
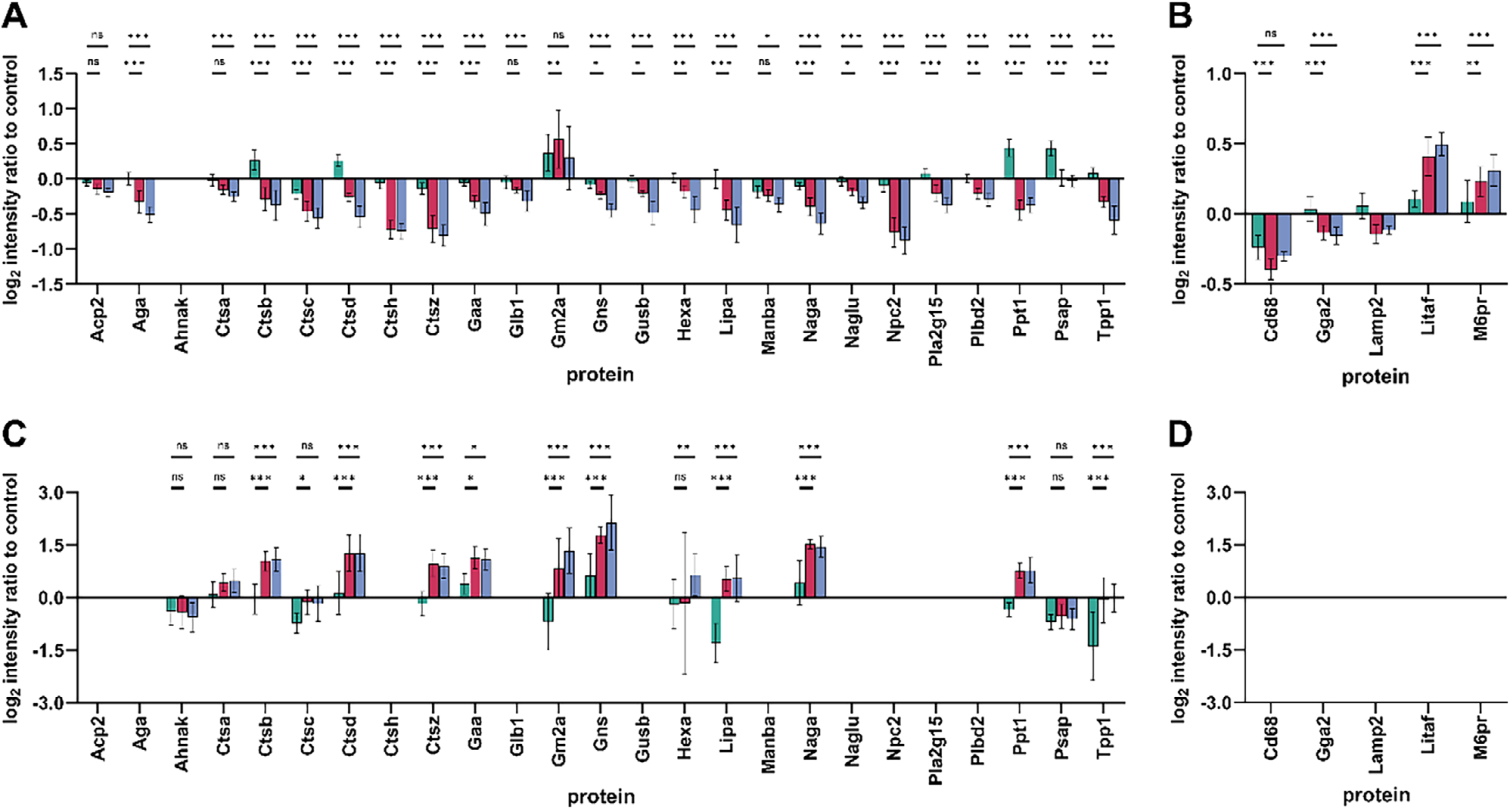
Significantly altered lysosomal proteins in NR8383 rat alveolar macrophages treated with Printex‐90 (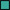), SiC nanofibers (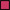), and Mitsui‐7 (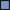) at 22.5 µg/mL for 18 h, relative to untreated controls. Differentially abundant proteins associated with the KEGG “lysosome” pathway are shown in the upper panels for cell lysates (A): luminal proteins; (B): lysosomal membrane‐associated proteins. The lower panels show corresponding proteins from cell culture supernatants (C): luminal proteins; (D): lacking lysosomal membrane‐associated proteins. Samples were generated in biological triplicate and measured three times using LC‐MS/MS. Results are expressed as mean values ± standard deviation. Statistical significance was determined by two‐way ANOVA (*p* ≤ 0.05) followed by Dunnett's multiple‐comparison test. Ns (not significant) *p* > 0.05; ^*^ *p* ≤ 0.05; ^**^ *p* ≤ 0.01; ^***^ *p* ≤ 0.001. Acp2: lysosomal acid phosphatase, Aga: aspartylglucosaminidase, Ahnak: ahnak nucleoprotein, Ctsa: cathepsin A, Ctsb: cathepsin B, Ctsc: cathepsin C, Ctsd: cathepsin D, Ctss: cathepsin S, Ctsz: cathepsin Z, Gaa: lysosomal alpha‐glucosidase, Glb1: beta‐galactosidase, Gm2a: GM2 ganglioside activator, Gns: N‐acetylglucosamine‐6‐sulfatase, Gusb: beta‐glucuronidase, Hexa: beta‐hexosaminidase subunit alpha, Lipa: lipase, Manba: beta‐mannosidase, Naga: alpha‐N‐acetylgalactosaminidase, Naglu: n‐acetyl‐alpha‐glucosaminidase, Npc2: NPC intracellular cholesterol transporter 2, Pla2g15: lysosomal phospholipase A and acyltransferase, Plbd2: putative phospholipase B‐like 2, Ppt1: palmitoyl‐protein thioesterase 1, Psap: prosaposin, Tpp1: tripeptidyl‐peptidase 1, Cd68: Cd68 molecule, Gga2: golgi associated, gamma adaptin ear containing, ARF binding protein 2, Lamp2: lysosome‐associated membrane glycoprotein 2, Litaf: lipopolysaccharide‐induced tumor necrosis factor‐alpha factor homolog, M6pr: cation‐dependent mannose‐6‐phosphate receptor.

Consequently, a focused analysis of significantly altered lysosomal proteins in whole cell lysates and cell culture supernatants was conducted to elucidate their involvement in this process. Figure 6 shows that the large majority of lysosomal proteins were reduced in their levels upon treatment with SiC NFs and Mitsui‐7 relative to controls in whole cell lysates, whereas the opposite was observed for cell culture supernatants.

**FIGURE 7:**
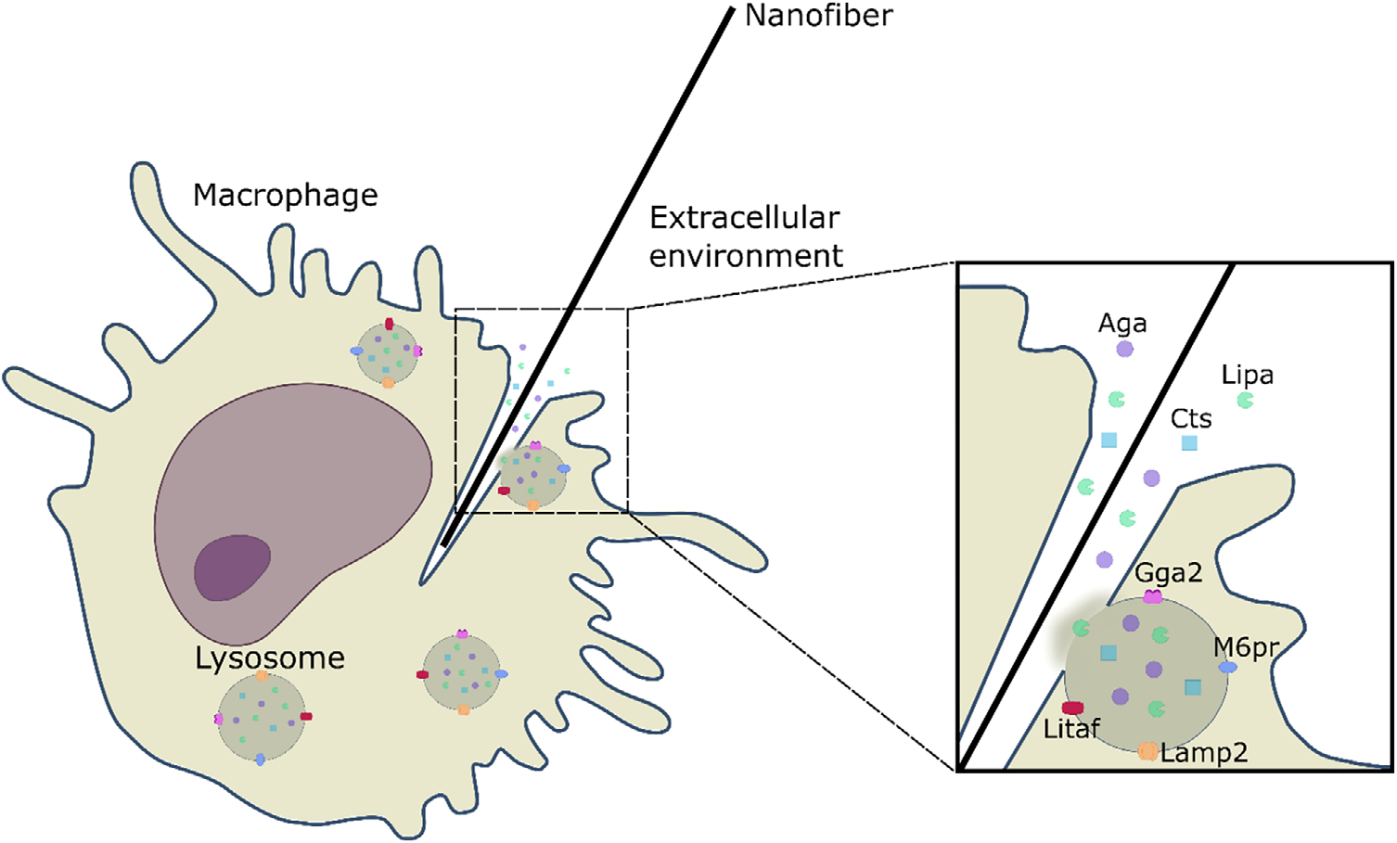
Release of lysosomal contents during frustrated phagocytosis in macrophages exposed to nanofibers. Aga: aspartylglucosaminidase, Cts: cathepsin A, B, C, D, H, Z, Gga2: golgi associated, gamma adaptin ear containing, ARF binding protein 2, Lamp2: lysosome‐associated membrane glycoprotein 2, Lipa: lipase, Litaf: lipopolysaccharide‐induced tumor necrosis factor‐alpha factor homolog, M6pr: cation‐dependent mannose‐6‐phosphate receptor. Modified from an illustration from NIAID NIH BIOART Source (https://bioart.niaid.nih.gov/).

The significantly altered proteins can be categorized based on cellular localization and relative abundance (Figure 6): Group A includes proteins in lysates that are located in the lysosomal lumen, such as six members of the cathepsin family (Supporting Information I: Table S4). Group B comprises proteins in lysates, which are located in the lysosomal membrane or associated with it (e.g., M6pr, Litaf). Group C includes proteins detected in the cell supernatants, which are typically located in the lysosomal lumen. Of note, no lysosomal membrane proteins were detected in cell culture supernatants (Figure 6D). This supports the initial hypothesis that lysosomal proteins are released into the extracellular environment as an early response to frustrated phagocytosis, occurring when lysosomes fuse with a phagosome that remains open, while the macrophage attempts to engulf an NF longer than its own length (Figure 7). This is supported by the fact, that acridine‐orange stained lysosomes have been directly observed to assemble at the membrane of open phagosomes and release their content early during frustrated phagocytosis of large deposits of lipoproteins and dead adipocytes [60, 61].

Individual protein levels support this hypothesis: M6pr, a membrane protein essential for trafficking acid hydrolases from the Golgi apparatus to lysosomes [62], is upregulated in macrophages exposed to NFs. The absence of M6pr in the supernatant suggests that it remains membrane‐bound and is not released from the lysosome, reinforcing the hypothesis of a lysosomal content loss from intact rather than degrading cells. This is also in line with the missing cytotoxicity in the presence of the low NF concentration used for proteome analysis. The increase in its level may be understood as a compensatory regulation of lysosomal enzymes lost from macrophages attempting to ingest and/or degrade persistent NFs. This mechanism likely underlies the release of luminal hydrolases to the external medium, such that cell lysates from treated macrophages show significantly reduced levels of luminal hydrolases. Notably, the majority of luminal proteins in Figure 6A are hydrolases (20 out of 25 proteins), including six peptidases of the cathepsin family (Ctsa, Ctsb, Ctsc, Ctsd, Ctsh, Ctsz), lipase (Lipa), and aspartylglucosaminidase (Aga), among others (Supporting Information I: Table S4).

Interestingly, a previous proteomics study also identified the decrease of cathepsins in lysates of differentiated THP‐1 macrophage‐like cells treated with fibrous materials [63]. Additionally, another study has also reported the release of Ctsb from macrophages treated with rigid, but not flexible, MWCNTs [64]. They proposed that the uptake of long and rigid fibers leads to cytosolic release of Ctsb, resulting from physical damage to lysosomes, specifically, the piercing of the lysosomal membrane. This leakage is thought to trigger NALP3‐dependent caspase‐1 activation, ultimately leading to cell death. However, this proposed mechanism does not align with the findings observed in the present study.

Similar to M6pr, the increase of Litaf (lysosomal‐associated transmembrane protein) can be explained by its active role in the cellular response to the NF. Litaf localizes on the cytoplasmic side of the lysosomes and is possibly upregulated by the NF treatment, since Litaf is involved in the activation of pro‐inflammatory cytokines, particularly TNF‐α, in macrophages [65]. Its increased abundance is in agreement with the elevated levels of secreted TNF‐α observed in the macrophage assay (Figure 1). Lamp2, a structural component of the lysosomal membrane, although slightly downregulated in cell lysates but not found in supernatants, further suggests that the membrane of lysosomes in cells treated with NFs remains stable (Figure 7). Similar to Lamp2, Gga2 levels decrease as an effect of the NF treatment, despite its cytoplasmic location. However, the reduction is minor in comparison to luminal proteins. Notably, Ctsa is a cathepsin partially associated with the membrane through complex formation with the lysosome‐associated membrane glycoprotein 2 (Lamp2) [59]. As a result, it exhibits an intermediate behavior, with levels between those of luminal proteins and M6pr, and its presence in the supernatant is much lower than for other cathepsins (Figure 7). Our hypothesis is further supported by the observation that the majority of lysosomal proteins whose levels remain unchanged relative to control (n = 27, Supporting Information I: Table S4) are predominantly associated with the lysosomal membrane. This pattern is consistent with a selective release of lysosomal luminal contents, arguing against a general lysosomal rupture or degradation.

Proteins that deviate from our hypothesis are Gm2a and CD68. The observed increase in Gm2a and decrease in CD68 levels could be due to their specific roles in lysosome dynamics, and further experiments are necessary to understand these changes. Although our findings primarily suggest a release of lysosomal contents into the extracellular environment following exposure to NFs, it is also plausible that, in addition, a lysosomal membrane permeabilization occurs, triggering pro‐inflammatory signaling cascades through the NALP3 inflammasome. Importantly, the activation of the NALP3 inflammasome is not the only consequence of NF toxicity, but the release of lysosomal content into the extracellular environment is part of a mechanism specific for pathogenic NFs. The mechanism we propose may not be limited to high‐aspect ratio materials. Based on a mechanistic confocal imaging study, Prashar and co‐workers showed that phagocytic cups of RAW 264.7 macrophages, when trying to ingest long filamentous bacteria, lose small fluorescent dextran molecules preloaded into lysosomes [66]. It was also shown that phagocytic cups became neither acidic nor did they accumulate lytic enzymes, suggesting a loss of lytic compounds from macrophages engulfing filamentous bacteria. Although inorganic fibers differ from the latter with respect to surface structure and rigidity, the loss of lytic enzymes from open membrane pouches may be a general mechanism. In the present work, we suggest that the process of frustrated phagocytosis of NFs is accompanied by the release of a characteristic pattern of lytic enzymes, which can be detected in the supernatant.

## 3 Conclusion

In this study, we provide the first direct evidence for the release of luminal lysosomal proteins from alveolar macrophages caused by putatively pathogenic nanofibers. This is most likely an early event associated with the frustrated phagocytosis of NFs. Macrophages cannot completely ingest NFs exceeding their own length, so that lysosomes fusing with nascent phagosomes will release their content into the open membrane pouches from where hydrolases, such as cathepsins, reach the extracellular space. This mechanism appears furthermore plausible because membrane and membrane‐associated lysosomal proteins were not extruded and remained unchanged in the cells. The non‐cytotoxic concentrations and the 18‐h incubation time also allowed alterations of other proteins linked to inflammation. Overall, we propose a proteomic signature that provides evidence for frustrated phagocytosis at a molecular level, including proteins predictive of morphology‐driven toxicity. This signature has been proven applicable to SiC, carbon NFs, and, in a previous study [30], also to TiO_2_ NFs, suggesting a morphology‐related, substance‐independent effect. Altogether, the proposed mechanism and the involved proteins offer a sensitive readout for assessing frustrated phagocytosis and NF pathogenicity through NAMs.

## 4 Experimental Section/Methods

### 4.1 Chemicals

Chemicals were purchased from: Carl Roth GmbH (Karlsruhe, Germany): citrate‐buffered 50% ethanol; Merck Millipore (Billerica, MA, USA): acetonitrile (MS grade), methanol (MS grade), sodium hydroxide; Sigma–Aldrich (Taufkirchen, Germany): actinomycin D, formic acid (≥ 95%), sodium azide, trichloroacetic acid (≥ 99.0%). Merck KGaA (Darmstadt, Germany): Roche cytotoxicity detection Kit Cat. No. 11644793001; Lipopolysaccharide (Cat. No. L8274); Triton X‐100 (MS‐grade): E‐Toxate test solution (E8904): Ham's F‐12 medium, Kaighn's modification (F‐12K) (Cat. No. N3520) Pan Biotech GmbH (Haidenach, Germany): Fetal bovine serum (Cat. No. P303302), penicillin/streptomycin concentrate (Cat. No. P06‐07100), phosphate buffered solution (Cat. No. P04‐36500), glutamine (Cat. No. P04‐80100).

### 4.2 Nanomaterials

Silicon carbide NFs (SiC) were sourced from ACS Material (Pasadena, CA, USA). Mitsui‐7 (Mitsui & Co., Japan) was provided by NRCWE under the designation JRCNM40011a. Printex‐90 was provided by Degussa‐Hüls (today Evonik, Germany). NMs were dispersed via sonication in accordance with the NANoREG D5.07 SOP 04 protocol (probe sonication, 16 min, 400 W, 10% amplitude, 0.05% (w/v) BSA‐water) [35]. Endotoxin testing using the Limulus Amebocyte Lysate Endochrome assay confirmed that all NMs were free of endotoxins.

### 4.3 Cell Culture of NR8383 Cells

Rat alveolar NR8383 macrophage (ATCC) were cultured as in Wiemann et al., 2016 [36]. Shortly, F‐12K medium was used, complemented with 2 mm glutamine, 100 U/mL penicillin, 10 µg/mL streptomycin, and 15% (v/v) fetal bovine serum (FBS) (all from PAN Biotech GmbH, Germany). Cells were grown in 500 mL flasks (Greiner Bio‐One, Germany, 175 cm^2^ with vented cap; 1.5 × 10^6^ cells/mL) at 37°C with 5% CO_2_ and passaged weekly. In general, for all replicates of the cytotoxicity assays, NR8383 cells were thawed and cultured under the described conditions. For all replicates of the assays, equally passaged and aged cells were harvested from the flasks and seeded in 96 well plates (3 × 10^5^ cells) as described in Section 4.5. A test for mycoplasma contamination was routinely performed on a regular basis.

### 4.4 Microscopy and Confocal Raman Microscopy

Bright‐field microscopic images (20x objective, Zeiss Axiovert 40C) of the NR8383 cells in the flasks were taken directly after the incubation period with the different materials. For Raman microscopy, cells were prepared as described in Vennemann et al., 2022 [67]. For Raman microscopy, cells were detached and resuspended by pipetting, and centrifuged onto glass slides using a cytocentrifuge (Shandon Cytospin 3) at 600 rpm for 6 min. After air‐drying, the cells were fixed with 4% phosphate‐buffered formaldehyde for 10 min, rinsed twice with phosphate‐buffered saline (PBS), and subsequently with distilled water. Brightfield microscopy combined with Raman imaging was performed using an Olympus BX43 microscope equipped with 60× water immersion objective (Olympus, N.A. 0.9). and a confocal Raman unit with LabSpec 6 software (Horiba Xplora Plus, HORIBA Jobin Yvon GmbH, Bensheim, Germany). Water immersion was essential to prevent sample damage during Raman laser exposure. A 532 nm laser (120 mW) was operated at 10% power with an acquisition time of 5 ms and an accumulation of 2 frames. Raman measurements were performed in the mapping mode on one representative cell of each material. The spectral ranges were recorded in a way that the respective characteristic peaks were included (Mitsui‐7: 1000–2800 cm^−1^, Printex‐90: 1000–3000 cm^−1,^ and SiC: 500–1200 cm^−1^). The areas for mapping of the three cells were selected so that almost a complete cell was included in each case. These areas covered a size of 285–432 µm^2^. No further data processing, such as smoothing or peak normalization, was performed. The shown Raman spectra for each material with characteristic bands were generated using data from one single‐point of the mappings.

### 4.5 Toxicity Screening Assays

Toxicity screening assays were performed as described in detail in Stobernack et al., 2025 [30]. These were based on the alveolar macrophage assay from Wiemann et al., 2016, and standard concentrations of the nanomaterials were applied [36]. This concentration range was often used in the alveolar macrophage assay because it may lead to a mean cellular concentration range of 15–120 pg per cell, if all particles/fibers settle and become completely ingested. Inhalation studies on rats have shown similar concentrations for alveolar macrophages loaded with poorly soluble [68]. Importantly, the approach allowed to demonstrate dose‐dependent effects of all testing materials and, furthermore, to identify a maximum non‐toxic concentration, which was necessary to detect protein responses hardly related to cell death. For toxicity screening, aqueous suspensions of test materials were diluted 1:1 in double‐concentrated serum‐free F‐12K medium (Merck, Germany) for cytotoxicity assays. Then they were further diluted to final concentrations of 180, 90, 45, and 22.5 µg/mL. NR8383 cells were seeded in 96‐well plates (3 × 10^5^ cells/well) and incubated in F‐12K medium with 5% FBS for 24 h. After removing supernatants, test materials were applied in serum‐free F‐12K medium (with glutamine and penicillin/streptomycin). Cells were exposed for 16 h for the LDH assay, β‐glucuronidase (GLU) assay, and TNF‐α production analysis, including cell‐free controls to account for material‐specific light interference. Although the signals from these controls were negligible, they were subtracted from the respective sample values to ensure accurate, interference‐corrected results. The LDH assay was used to evaluate cellular membrane permeability and, consequently, cell viability. The second assay measures the activity of GLU, an enzyme predominantly found in lysosomes. Its activity serves as an indicator of macrophage activation and/or lysosomal integrity. Finally, the release of TNF‐α offers a measurement for assessing inflammatory processes.

#### 4.5.1 Lactate Dehydrogenase and Glucuronidase Assay

LDH and GLU release from treated and untreated NR8383 cells was measured as described by Wiemann et al., 2016 using three biological replicates with three technical replicates each [36]. In each experiment, 200 µL of cell culture supernatant was collected from control and exposed cells after 16 h incubation and centrifuged (10 min, 140 g, room temperature (RT)). For LDH, 50 µL of supernatant was incubated with the Roche Cytotoxicity Kit reaction mix (Cat. No. 11644793001, Roche, Germany) per the manufacturer's protocol. The reaction was stopped after 10 min by adding 50 µL 1N HCl, and optical density (OD) was measured at 492/620 nm. To determine GLU activity, 50 µL of the supernatant was incubated at 37°C with 50 µL of GLU reaction mix (per plate: 5 mL 0.1 m sodium acetate buffer, pH 5, 1 mL 13.3 mm p‐nitrophenyl‐D‐glucuronide, Cat. No N1627, Sigma–Aldrich, Germany, and 1 mL 1% Triton‐X‐100). The reaction was stopped after 90 min by the addition of 50 µL 0.2 m NaOH (Merck KGaA, Germany). OD was measured at 405/620 nm for LDH and at 405 nm for GLU measurement in a plate photometer (Tecan 200Pro, Tecan, Germany). Activities were expressed relative to the 100% value of Triton X‐100‐lysed NR8383 cells (positive control). Results were corrected for cell‐free adsorption by preparing individual cell‐free blanks for each particle/fiber concentration using supernatants from wells that received identical material concentrations but no cells. Calculations were made according to (OD Sample_(C1–C4)_—OD Cell‐free_(C1–C4)_)/(OD Triton—OD Blank) × 100, where C1–C4 represent the four particle/fiber concentrations.

#### 4.5.2 Tumor Necrosis Factor Alpha

Release of TNF‐α was determined in supernatants of cell in culture after centrifugation and measured by a specific enzyme‐linked immunosorbent assay (ELISA) for rat TNF‐α (ELISA MAX Deluxe Set Rat TNF‐alpha, Cat. No. BLD‐438204, BioLegend, San Diego, United States of America). 0.5 µg/mL LPS (Merck, Germany) was used as a positive control.

### 4.6 Sample Preparation for Proteomics

5.5 × 10^6^ NR8383 macrophages were seeded into 25 cm^2^ cell flasks (Greiner, Germany) and exposed in three biological replicates (cells of different passage number, exposed to freshly prepared material dispersions) to treatment concentrations of 22.5 µg/mL in serum free F‐12K for 18 h (37°C, 5% CO_2_). This concentration was selected based on the absence of cytotoxic effects at this dose, ensuring that observed responses reflect fiber‐specific effects rather than secondary consequences of cell death (Figure 1). Harvested cells were suspended for counting in a Coulter Counter Z2 (Beckman Coulter, Indianapolis, USA). Cell suspension (200 µL) was taken for cytospin preparations (Cytospin 3, Shandon). Remaining cells were centrifuged (5 min, 200 x g) and supernatants were collected. All supernatants were concentrated using 3 kDa Amicon Ultra (Merck Millipore, Billerica, MA, USA) centrifugal filters by spinning at 4°C at 5000 × g until the volume was reduced to approximately 250 µL. Cells were lysed, and proteins were extracted and digested using the PreOmics iST‐NHS kit (PreOmics, Planegg/Martinsried, Germany) following the manufacturer's guidelines. Protein levels were quantified with a BCA assay kit (Cayman Chemicals, Ann Arbor, MI, USA). Dried peptides from supernatants were reconstituted with 0.1% (v/v) trifluoroacetic acid (TFA), 5% (v/v) acetonitrile, and water to achieve a final peptide concentration of 50 ng/µL (total volume 200 µL) for LFQ measurement.

#### 4.6.1 TMT Labeling

Cell lysate digests were labeled with TMT6‐plex reagents following the protocol described by Stobernack et al. [41]. Briefly, TMT6‐plex labels (0.8 mg; Thermo Fisher, Bremen, Germany) were equilibrated at RT for 5 min, dissolved in 41 µL anhydrous acetonitrile for 5 min, vortexed thoroughly, and subjected to a brief centrifugation (10 s). A volume of 4.1 µL (80 µg) of the TMT6‐plex label was then added to 10 µg of protein digest diluted in 18 µL of 0.1 mol/L triethylammonium bicarbonate (TEAB). The labeling reaction was incubated for 1 h at RT, shaking at 300 rpm, and quenched (15 min) by adding 1.5 µL of 5% hydroxylamine solution. The labeled peptides were acidified with 220 µL of 0.1% formic acid in water and purified using the PreOmics iST‐NHS kit according to the manufacturer's protocol. Equal labeled peptide amounts of each sample were combined, dried under vacuum at 40°C for 2.5 h, and reconstituted with 0.1% (v/v) trifluoroacetic acid (TFA), 5% (v/v) acetonitrile, and water, resulting in a final peptide concentration of 50 ng/µL.

### 4.7 LC‐MS/MS Measurements

Samples were analyzed in technical triplicates using a reverse‐phase nano‐LC system (Ultimate 3000 RLSCnano, Thermo Scientific, USA) coupled to an Orbitrap Q Exactive Plus mass spectrometer (Thermo Scientific) operated via a Nanospray Flex ion source with a stainless‐steel emitter. For each run, 400 ng of peptide material was loaded. Peptides were captured on a trap column (Acclaim PepMap 100 C18, 3 µm, 100 Å, 75 µm i.d. × 2 cm, Thermo Scientific) pre‐equilibrated with 0.05% (v/v) TFA and 2% (v/v) acetonitrile in water at 45°C for 5 min. Subsequently, the trap column was switched in line with the analytical column (Acclaim PepMap 100 C18, 2 µm, 100 Å, 75 µm i.d. × 50 cm, Thermo Scientific), and chromatographic separation was carried out at 350 nL/min.

Elution of peptides was achieved using a linear gradient from 5.7% to 35.2% solvent B over 90 min, followed by an increase to 50% solvent B over an additional 5 min. Solvent A consisted of 0.1% (v/v) formic acid in water, and solvent B consisted of 0.1% (v/v) formic acid in 80% (v/v) acetonitrile. Mass spectra were acquired in data‐dependent acquisition (DDA) mode with higher‐energy collisional dissociation (HCD) fragmentation. Full MS survey scans were recorded at a resolution of 70 000 over an *m/z* range of 350–1500. For LFQ samples (supernatants), MS/MS scans were acquired for the 10 most intense precursor ions using an isolation window of 1.6 *m/z* and a resolution of 17 500. For TMT samples (cell lysates), the 12 most intense precursor ions were selected using an isolation window of 1.2 *m/z* and a resolution of 35 000. Dynamic exclusion was set to 30 s, and the automatic gain control (AGC) target was 3 × 10^6^. To ensure system stability and performance during data acquisition, HeLa digest (Thermo Scientific, USA, Cat.Nr. 88329) quality control samples were regularly injected throughout the LC‐MS/MS sequence. The Orbitrap mass analyzer was calibrated weekly according to the manufacturer's recommendations. Additional LC‐MS parameters are detailed in Supporting Information I: Table S5.

### 4.8 Protein Identification and Data Analysis

MaxQuant (version 2.4.2.0; Max‐Planck‐Institute of Biochemistry, Planegg, Germany) was used for analysis of MS and MS/MS spectra from each LC‐MS run [69]. Protein identification was performed with the integrated Andromeda search engine against the *Rattus norvegicus* reference proteome (UniProt ID: UP000002494, release February 5, 2022). Database searches were conducted using a precursor mass tolerance of 4.5 ppm and a fragment mass tolerance of 20 ppm. Methionine oxidation and protein N‐terminal acetylation were set as variable modifications, while carbamidomethylation of cysteine was defined as a fixed modification. Peptides containing more than two missed cleavages or fewer than seven residues were excluded. The FDR for both peptide‐ and protein‐level identifications was controlled at 1%. For label‐free datasets, normalization was performed automatically in MaxQuant using LFQ normalization, while TMT data were normalized to a pooled reference channel containing an equal mixture of all samples in the experiment. TMT impurity correction factors provided by the manufacturer were included in the MaxQuant analysis. Further MaxQuant parameters are given in Supporting Information I: Table S6.

### 4.9 Statistical Analysis

For pre‐processing of proteomic analysis, log_2_‐transformed protein intensities were calculated after removing reverse hits, potential contaminants, and proteins identified only by site. Data were reported as mean values ± standard deviation. Each experiment included three biological replicates per sample, with each biological replicate analyzed in technical triplicates. To enhance robustness, only proteins with at least 70% valid values in at least one experimental group were retained. Remaining missing values were imputed in Perseus using the standard approach of sampling from a downshifted and width‐compressed normal distribution (width = 0.3; downshift = 1.8 standard deviations) to approximate values below the detection limit. Statistical significance (*p* ≤ 0.05) between sample groups was assessed using a two‐way analysis of variance (ANOVA) followed by dunnett's test (for detailed results see Supporting Information II Tables S9–S12). Comparisons between treatments were only within datasets generated via the same quantification technique (either TMT or LFQ). Significant differences in protein abundance were determined using Perseus by applying a permutation‐based two‐sample *t*‐test (S_0_ = 0.1, FDR ≤ 0.05, 250 randomizations), and visualized in a volcano plot. Comparative analyses between treated and untreated samples were conducted using Perseus software version 2.1.0.0 (Max‐Planck‐Institute of Biochemistry, Planegg, Germany) [70], and Prism version 10.1.2 (GraphPad Software, San Diego, CA, USA). Data filtering, statistical calculations, and visualizations were performed using Perseus, Prism, and Excel (Office Professional 2021 Plus, Microsoft, Redmond, WA, USA). Pathway enrichment analysis was done using the KEGG database through STRING DB (species: *Rattus norvegicus*, background: whole genome) [71]. STRING DB conducts enrichment based on a Fisher's exact (hypergeometric) test and applies Benjamini–Hochberg correction for multiple comparisons [71].

## Author Contributions

Conceptualization was performed by T.S., A.H., M.P., and V.I.D. Methodology was developed by T.S., A.V., J.S., O.G., R.L., M.W., and V.I.D. Software development was carried out by T.S., A.V., and C.R. Formal analysis was conducted by T.S., A.V., and V.I.D. Investigation was performed by T.S., A.V., M.P., and V.I.D. Resources were provided by A.H. and M.W. Data curation was carried out by T.S. The original draft of the manuscript was prepared by T.S. and V.I.D., and the manuscript was reviewed and edited by T.S., A.V., C.R., J.S., O.G., R.L., M.P., A.H., M.W., and V.I.D. Visualization was performed by T.S., A.V., and C.R. Supervision was provided by A.H., M.W., and V.I.D. Project administration was carried out by A.H. and V.I.D., and funding was acquired by A.H. and V.I.D. All authors have read and agreed to the published version of the manuscript.

## Funding

This work was supported by the EU H2020 project HARMLESS (grant agreement No 953183), and by the BfR project 1322‐777.

## Conflicts of Interest

The authors declare no conflicts of interest.

## Supporting information




**Supporting File 1**: smll72163‐sup‐0001‐SuppMat.pdf.

**Supporting File 2**: smll72163‐sup‐0002‐Supplement_II‐Table S1–S12.xlsx.

## Data Availability

The data that support the findings of this study are available from the corresponding author upon reasonable request.
