# Supplementary material for: Proteomic Profiling of Alveolar Macrophages Identifies Loss of Lysosomal Content as an Indicator of Nanofiber‐Induced Frustrated Phagocytosis
Source: Small. 2026 Jan 17;22(11):e10530. doi: 10.1002/smll.202510530 (PMC12921543; doi:10.1002/smll.202510530)
Supplement: Supplementary file 1 — Supporting File 1: smll72163‐sup‐0001‐SuppMat.pdf. [file SMLL-22-e10530-s001.pdf]

Supplementary material I

Proteomic Profiling of Alveolar Macrophages Identifies Loss of Lysosomal Content as an Indicator of Nanofiber-Induced Frustrated Phagocytosis

Tobias Stobernack, Antje Vennemann, Carla Ribalta, Julia Schendel, Rico Ledwith, Mario Pink, Andrea Haase, Martin Wiemann, Verónica I. Dumit*

T. Stobernack, C. Ribalta, J. Schendel, R. Ledwith, M. Pink, A. Haase, V.I. Dumit

German Federal Institute for Risk Assessment (BfR), Department of Chemical and Product Safety, Max-Dohrn-Straße 8-10, 10589 Berlin, Germany

*Corresponding author e-mail: Veronica.Dumit@bfr.bund.de

A. Vennemann, M. Wiemann

IBE R&D Institute for Lung Health gGmbH, Mendelstraße 11, 48149 Münster, Germany

C. Ribalta

Federal Institute for Occupational Safety and Health (BAuA), Materials and Particulate Hazardous Substances, Nöldnerstraße 40-42, 10317 Berlin, Germany

A. Haase

Freie Universität Berlin, Institute of Pharmacy, Berlin, Germany

Figures:

Figure S1. Identified proteins of the preliminary fingerprint 3

Tables:

Table S1. KEGG pathways via STRING DB (FDR≤ 0.01) associated to the significantly altered proteins in lysates of NR8383 cells treated with Printex-90, SiC nanofiber or Mitsui-7 (22.5 µg/mL, 18 h). 4

Table S2. KEGG pathways via STRING DB (FDR≤ 0.01) associated to the significantly altered proteins in supernatants of NR8383 cells treated with Printex-90, SiC nanofiber or Mitsui-7 (22.5 µg/mL, 18 h). 5

Table S3: List of 58 potential biomarker for morphology-driven NF toxicity included in the preliminary fingerprint. 6

Table S4: Lysosomal proteins detected by the proteomic measurements of cell lysates or cell culture supernatant. 8

Table S5. NanoUHPLC and mass spectrometric parameters. 9

Table S6. MaxQuant database search parameters. 10

Figures

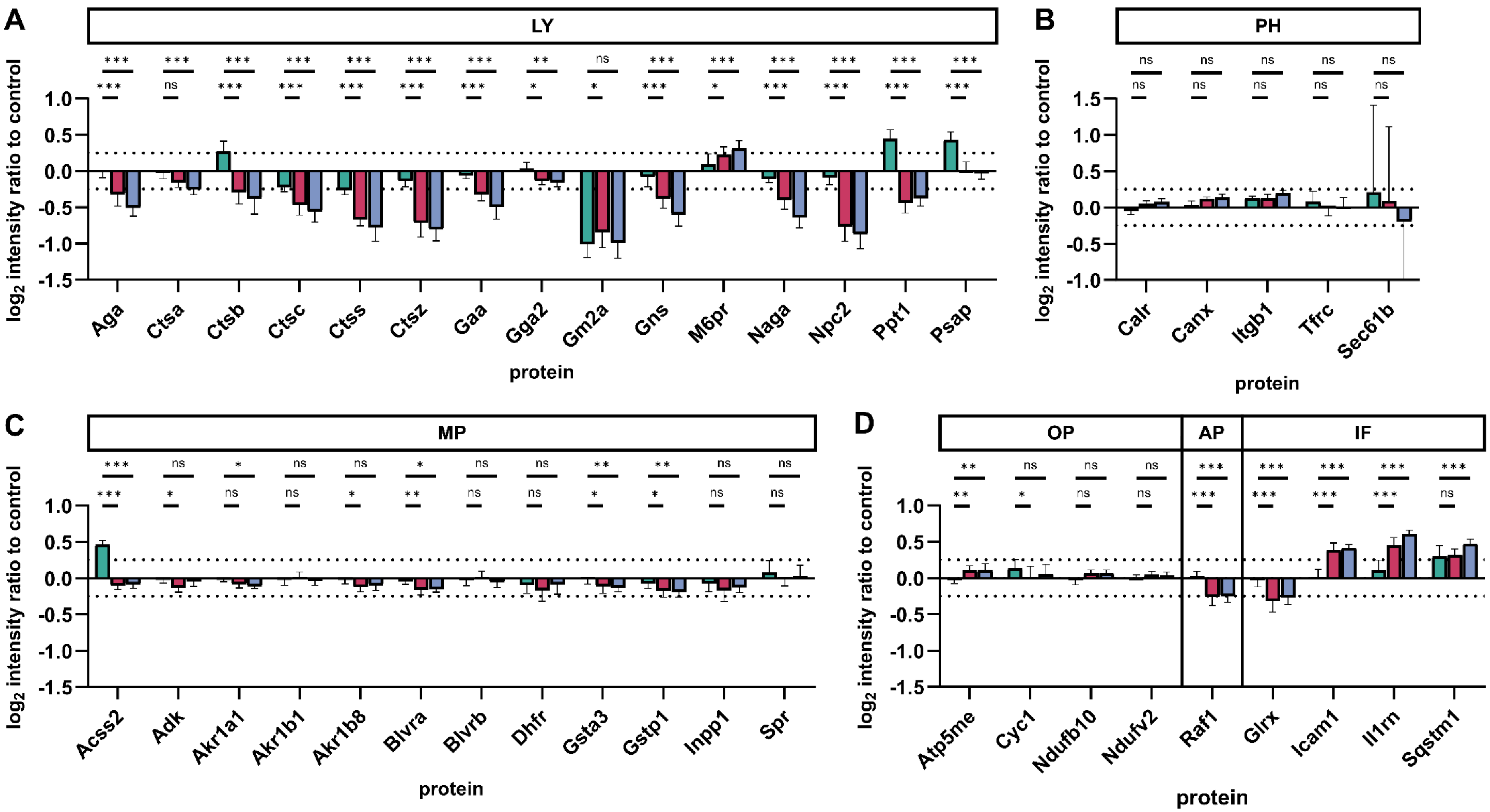

Figure S1. Detected proteins from the preliminary fingerprint in NR8383 rat alveolar macrophages treated with Printex-90 (■), SiC nanofibers (■) and Mitsui-7 (■) at 22.5 µg/mL for 18 h. 41 of the 58 previously identified fingerprint proteins were detected in cell lysates. AP: apoptosis, IF: inflammation, LY: lysosome, MP: metabolism pathways, OP: oxidative phosphorylation, PH: phagosome

Tables

Table S1. KEGG pathways via STRING DB (FDR≤ 0.01) associated to the significantly altered proteins in lysates of NR8383 cells treated with Printex-90, SiC nanofiber or Mitsui-7 (22.5 µg/mL, 18 h).

material	KEGG pathway description	count	FDR
Printex-90	Lysosome	8 of 120	4,7E-06
	Metabolic pathways	16 of 1431	4,7E-03
	Fatty acid metabolism	4 of 57	6,5E-03
SiC	Lysosome	30 of 120	1,4E-29
	Metabolic pathways	41 of 1431	8,8E-09
	Tuberculosis	13 of 155	4,3E-07
	Glycosaminoglycan degradation	6 of 18	4,6E-06
	Carbon metabolism	10 of 112	9,7E-06
	Cholesterol metabolism	7 of 48	3,2E-05
	Citrate cycle (TCA cycle)	6 of 30	3,4E-05
	Galactose metabolism	5 of 25	2,6E-04
	Parkinson disease	11 of 235	5,1E-04
	Glycolysis / Gluconeogenesis	6 of 58	7,5E-04
	Other glycan degradation	4 of 17	9,8E-04
	Ferroptosis	5 of 38	1,1E-03
	Apoptosis	7 of 122	4,1E-03
	Autophagy - animal	7 of 133	6,3E-03
	Pyruvate metabolism	4 of 35	8,7E-03
Mitsui-7	Lysosome	35 of 120	2,4E-31
	Metabolic pathways	56 of 1431	2,0E-11
	Carbon metabolism	14 of 112	8,1E-08
	Citrate cycle (TCA cycle)	8 of 30	1,7E-06
	Other glycan degradation	6 of 17	2,1E-05
	Glycosaminoglycan degradation	6 of 18	2,4E-05
	Tuberculosis	11 of 155	4,2E-04
	Parkinson disease	13 of 235	7,2E-04
	Galactose metabolism	5 of 25	1,2E-03
	Ribosome	10 of 158	1,9E-03
	Glycolysis / Gluconeogenesis	6 of 58	4,7E-03
	Phagosome	9 of 149	5,1E-03
	Ferroptosis	5 of 38	5,1E-03
	Central carbon metabolism in cancer	6 of 62	5,1E-03
	Apoptosis	8 of 122	5,5E-03
	Oxidative phosphorylation	8 of 128	7,0E-03
	Fluid shear stress and atherosclerosis	8 of 134	8,8E-03

Table S2. KEGG pathways via STRING DB (FDR≤ 0.01) associated to the significantly altered proteins in supernatants of NR8383 cells treated with Printex-90, SiC nanofiber or Mitsui-7 (22.5 µg/mL, 18 h).

material	KEGG pathway description	count	FDR
SiC	Ribosome	10 of 158	4,3E-06
	Lysosome	7 of 120	5,9E-04
Mitsui-7	Ribosome	16 of 158	7,7E-08
	Metabolic pathways	46 of 1431	1,4E-07
	Pyruvate metabolism	9 of 35	1,9E-07
	Carbon metabolism	13 of 112	2,2E-07
	Protein processing in endoplasmic reticulum	14 of 158	1,0E-06
	Prion disease	16 of 260	9,4E-06
	Antigen processing and presentation	9 of 69	1,5E-05
	Salmonella infection	13 of 207	8,3E-05
	Proteasome	7 of 47	1,1E-04
	Glycolysis / Gluconeogenesis	7 of 58	3,5E-04
	Lysosome	9 of 120	6,3E-04
	Cysteine and methionine metabolism	6 of 46	8,5E-04
	Glyoxylate and dicarboxylate metabolism	5 of 27	8,5E-04
	Parkinson disease	12 of 235	8,5E-04
	Citrate cycle (TCA cycle)	5 of 30	1,0E-03
	Propanoate metabolism	5 of 30	1,0E-03
	Legionellosis	6 of 50	1,0E-03
	Huntington disease	13 of 289	1,1E-03
	Amyotrophic lateral sclerosis	14 of 346	1,6E-03
	Alzheimer disease	14 of 352	1,7E-03
	Viral carcinogenesis	10 of 185	1,7E-03
	Adherens junction	6 of 63	2,3E-03
	NOD-like receptor signaling pathway	9 of 160	2,4E-03
	Biosynthesis of amino acids	6 of 76	5,3E-03
	HIF-1 signaling pathway	7 of 107	5,3E-03
	Phagosome	8 of 149	6,7E-03
	Gap junction	6 of 81	6,7E-03
	Endocytosis	10 of 234	7,2E-03
	Fc gamma R-mediated phagocytosis	6 of 84	7,4E-03
	RNA transport	8 of 158	8,3E-03
	Estrogen signaling pathway	7 of 122	8,6E-03

Table S3: List of 58 potential biomarker for morphology-driven NF toxicity included in the preliminary fingerprint, and the corresponding correlation with present results obtained by exposing NR8383 macrophages with half the concentration (22,5 µg/mL) for Printex-90, SiC NFs, and Mitsui-7.

#	Uniprot ID	Protein names	Abbreviation	Trend	Confirms the fingerprint
1	P30919	Aspartylglucosaminidase	Aga	down	+
2	Q6AYS3	Cathepsin A	Ctsa	down	+
3	P00787	Cathepsin B	Ctsb	down	+
4	P80067	Cathepsin C	Ctsc	down	+
5	Q02765	Cathepsin S	Ctss	down	+
6	Q9R1T3	Cathepsin Z	Ctsz	down	+
7	Q6P7A9	Lysosomal alpha-glucosidase	Gaa	down	+
8	G3V8F7	Golgi associated, gamma adaptin ear containing, ARF binding protein 2	Gga2	down	-
9	Q6IN37	GM2 ganglioside activator	Gm2a	down	-
10	Q32KJ5	N-acetylglucosamine-6-sulfatase	Gns	down	+
11	Q6AY20	Cation-dependent mannose-6-phosphate receptor	M6pr	up	+
12	Q66H12	Alpha-N-acetylgalactosaminidase	Naga	down	+
13	F7FJQ3	NPC intracellular cholesterol transporter 2	Npc2	down	+
14	P45479	Palmitoyl-protein thioesterase 1	Ppt1	down	+
15	P10960	Prosaposin	Psap	down	-
16	Q99376	Transferrin receptor protein 1	Tfrc	up	-
17	P18418	Calreticulin	Calr	up	-
18	B2RZD1	Protein transport protein Sec61 subunit beta	Sec61b	up	-
19	P49134	Integrin beta-1	Itgb1	up	-
20	P35565	Calnexin	Canx	up	-
21	A0A0G2K1E2	Integrin subunit alpha 5	Itga5	up	n.d.
22	Q5XI22	acetyl-CoA acetyltransferase, cytosolic	Acat2I1	down	n.d.
23	G3V9U0	propionate--CoA ligase	Acss2	down	-
24	Q64640	adenosine kinase	Adk	down	-
25	P51635	aldo-keto reductase family 1 member A1	Akr1a1	down	-
26	P07943	aldo-keto reductase family 1 member B1	Akr1b1	down	-
27	G3V786	aldo-keto reductase family 1 member B10	Akr1b8	down	-
28	P46844	biliverdin reductase A	Blvra	down	-
29	A6J9C6	biliverdin reductase B	Blvrb	down	-
30	B5DFG6	carbonic anhydrase	Car13	down	n.d.
31	Q7TP52	carboxymethylenebutenolidase homolog	Cmbl	down	n.d.

32	Q920D2	dihydrofolate reductase	Dhfr	down	-
33	P18645	UDP-glucose 4-epimerase	Gale	down	n.d.
34	G3V960	guanidinoacetate N-methyltransferase	Gamt	down	n.d.
35	P19468	glutamate--cysteine ligase catalytic subunit	Gclc	down	n.d.
36	P04904	glutathione S-transferase alpha-3	Gsta3	down	-
37	P04906	glutathione S-transferase P	Gstp1	down	-
38	Q32PY9	probable gluconokinase	Idnk	down	n.d.
39	A6INV6	inositol-1,4-bisphosphate 1-phosphatase	Inpp1	down	-
40	P0C548	patatin-like phospholipase domain-containing protein 2	Pnpla2	down	n.d.
41	M0R7I5	uncharacterized protein	Sgsh	down	n.d.
42	P18297	sepiapterin reductase	Spr	down	-
43	Q62867	gamma-glutamyl hydrolase	Ggh	down	n.d.
44	P29419	ATP synthase subunit e, mitochondrial	Atp5me	up	-
45	Q76MV3	cytochrome C oxidase assembly protein COX17	Cox17	up	n.d.
46	D3ZFQ8	cytochrome c-1	Cyc1	up	-
47	D4A0T0	NADH dehydrogenase [ubiquinone] 1 beta subcomplex subunit 10	Ndufb10	up	-
48	F1LPG5	NADH dehydrogenase [ubiquinone] 1 beta subcomplex subunit 4	Ndufb4	up	n.d.
49	P19234	NADH dehydrogenase [ubiquinone] flavoprotein 2, mitochondrial	Ndufv2	up	-
50	P55213	Caspase-3	Casp3	down	n.d.
51	P11345	RAF proto-oncogene serine/threonine-protein kinase	Raf1	down	+
52	O08623	sequestosome-1	Sqstm	up	+
53	P07824	Arginase-1	Arg1	up	n.d.
54	P25086	interleukin-1 receptor antagonist protein	Il1rn	up	+
55	Q00238	intercellular adhesion molecule 1	Icam1	up	+
56	Q62625	microtubule-associated proteins 1A/1B light chain 3B	Map1lc3b	up	n.d.
57	Q63264	interleukin-1 beta	Il1b	down	n.d.
58	Q9ESH6	glutaredoxin-1	Glrx	down	+

Proteins marked with “+” align with the original preliminary fingerprint, those marked with “-” exhibit different trend or low sensitivity compared to the previous work, and those marked “n.d.” were not detected here.

Table S4: Lysosomal proteins detected by the proteomic measurements of cell lysates (L.) or cell culture supernatant (S.), including those significantly altered by SiC nanofibers and Mitsui-7 treatments, and those that were not affected. Proteins marked in orange are membrane-bound or face the cytoplasmic side of the lysosome, while those in green are luminal proteins. * indicates if the protein has a hydrolase function; ↑ and ↓ indicate if protein levels are increased or decreased, respectively.

**altered levels for NF treatment**			**non-altered levels**
ID	L.	S.	ID
Acp2*	↓		Ap1b1
Aga*	↓		Ap1m1
Ahnak		↓	Ap1s1
Cd68	↓		Ap3b1
Ctsa*	↓	↑	Ap3d1
Ctsb*	↓	↑	Ap3m1
Ctsc*	↓	-	Asah1*
Ctsd*	↓	↑	Atp6ap1
Ctsh*	↓		Atp6v0a1
Ctsz*	↓	↑	Atp6v0d1
Gaa*	↓	↑	Atp6v0d2
Gns	↓	↑	Cd63
Gga2	↓		Clta
Glb1*	↓		Cltb
Gm2a	↑	↑	Cltc
Gusb*	↓		Fuca1*
Hexa*	↓	↑	Gba*
Lamp2	↓		Igf2r
Lipa*	↓	↑	Lamp1
Litaf	↑		Lgmn*
M6pr	↑		Napsa*
Manba*	↓		Npc1
Naga*	↓	↑	Plaa*
Naglu*	↓		Scarb2
Npc2	↓		Slc11a1
Pla2g15*	↓		Sumf1
Plbd2*	↓		Tcirg1
Ppt1*	-	↑	
Psap*	-	↓	
Tpp1*	↓	-	

Table S5. NanoUHPLC and mass spectrometric parameters.

**parameter**	**value**	
trapping eluent	2% (v/v) CH_3CN, 0.05% (v/v) TFA, H_2O	
trapping flow [µL/min]	5	
trapping time [min]	5	
eluent A	0.1% (v/v) HCOOH, H_2O	
eluent B	80% (v/v) CH_3CN, 0.1% (v/v) HCOOH, H_2O	
flow [µL/min]	0.350	
gradient program	0 min 5.7%B	
	5 min 5.7%B	
	95 min 35.2%B	
	100 min 50%B	
	102 min 98%B	
	107 min 98%B	
	108 min 5.7%B	
	120 min 5.7%B	
source voltage [V]	2,000	
capillary temperature [°C]	300	
S-Lens RF Level	50	
	TMT	LFQ
m/z range	350 - 1500	350 - 1500
resolution (at *m/z* 200)	70,000	70,000
microscans	1	1
AGC target	$3*10^6$	$3*10^6$
maximum ion injection time (IT) [ms]	50	50
number of scan ranges	1	1
spectrum data type	profile	profile
resolution (at *m/z* 200)	35,000	17,500
microscans	1	1
AGC target	$1*10^5$	$1*10^5$
maximum ion injection time (IT) [ms]	100	50
loop count	12	10
top N	12	10
MSX count	1	1
MSX isochronous ITs	-	-
isolation window [*m/z*]	1.2	1.6
isolation offset [*m/z*]	0.0	0
fixed first mass [*m/z*]	100	-
NCE	32	28
spectrum data type	profile	profile
minimum AGC target	$1.6*10^3$	$1.6*10^3$
intensity threshold	$1.6*10^4$	$3.2*10^4$
charge exclusion	unassigned, 1, 8, >8	unassigned, 1, >8
peptide match	preferred	preferred
dynamic exclusion time [s]	30.0	60.0

Table S6. MaxQuant database search paramaters.

Parameter	Value
Version	2.4.2.0
Include contaminants	TRUE
PSM FDR	0.01
PSM FDR Crosslink	0.01
Protein FDR	0.01
Site FDR	0.01
Use Normalized Ratios for Occupancy	Normalized ratios
Min. peptide Length	7
Min. score for unmodified peptides	0
Min. score for modified peptides	40
Min. delta score for unmodified peptides	0
Min. delta score for modified peptides	6
Min. unique peptides	0
Min. razor peptides	1
Min. peptides	1
Use only unmodified peptides	TRUE
Mods. included in protein quantification	Oxidation (M); Acetyl (Protein N-term)
Peptides used for protein quantification	Unique + Razor
Discard unmodified counterpart peptides	TRUE
Label min. ratio count	2
Use delta score	FALSE
iBAQ	FALSE
iBAQ log fit	FALSE
Match between runs	TRUE
Matching time window [min]	0.4
Alignment time window [min]	20
Alignment ion mobility window [indices]	1
Find dependent peptides	FALSE
Fasta file	*Rattus norvegicus* (UP000002494, February 5th, 2022)
Decoy mode	revert
Include contaminants	TRUE
Advanced ratios	TRUE
Second peptides	TRUE
Stabilize large LFQ ratios	TRUE
Separate LFQ in parameter groups	FALSE
Require MS/MS for LFQ comparisons	TRUE
Calculate peak properties	FALSE
Main search max. combinations	200
Advanced site intensities	TRUE
Max. peptide mass [Da]	4600
Min. peptide length for unspecific search	8
Max. peptide length for unspecific search	25
Razor protein FDR	TRUE
Max mods in site table	3
Match unidentified features	FALSE
Evaluate variant peptides separately	TRUE
Label min ratio count	2
Variation mode	None
Instrument Type	Orbitrap
Main search peptide tolerance	4.5 ppm
Intensity determination	Value at maximum
FTMS MS/MS match tolerance	20 ppm
First search peptide tolerance	20 ppm

Table S6 (continued).

Parameter	Value
Top MS/MS peaks per Da interval. (FTMS)	12
Da interval. (FTMS)	100
MS/MS deisotoping (FTMS)	TRUE
MS/MS deisotoping tolerance (FTMS)	7
MS/MS deisotoping tolerance unit (FTMS)	ppm
MS/MS higher charges (FTMS)	TRUE
MS/MS water loss (FTMS)	TRUE
MS/MS ammonia loss (FTMS)	TRUE
MS/MS dependent losses (FTMS)	TRUE
MS/MS recalibration (FTMS)	FALSE
